# Percutaneous transhepatic treatment of a unique portal vein malformation with portal hypertension in a pediatric patient

**DOI:** 10.1186/s42155-021-00239-1

**Published:** 2021-06-07

**Authors:** Paolo Marra, Ludovico Dulcetta, Claudia Pellegrinelli, Lorenzo D’Antiga, Sandro Sironi

**Affiliations:** 1 Department of Radiology - Papa Giovanni XXIII Hospital, University of Milano Bicocca, Piazza OMS 1, 24127 Bergamo, Italy; 2 Paediatric Hepatology, Gastroenterology, and Transplantation - Papa Giovanni XXIII Hospital, Bergamo, Italy

**Keywords:** Portal vein malformation, Percutaneous transhepatic portography, Angioplasty

## Abstract

**Background:**

Anomalies of the portal venous system can be congenital or acquired, the latter being related to spontaneous thrombosis or iatrogenic alterations such as complications of perinatal catheterization of the umbilical vein. These conditions can be clinically silent for years and then manifest abruptly causing severe clinical emergencies.

**Case presentation:**

This case report describes the diagnosis and interventional management of a singular abnormality in the portal venous system of an 8-year-old female that led to severe portal hypertension and acute variceal bleeding. Peculiar imaging findings were not pathognomonic for any of the known congenital and acquired portal vein anomalies: absence of a normal extrahepatic portal vein; splenic and mesenteric veins merging into a dilated left gastric vein; presence of an aberrant mesenteric venous collateral with a stenotic connection with the intrahepatic right portal branch; and absence of porto-systemic shunt. The case was successfully managed with percutaneous transhepatic portography and angioplasty.

**Conclusions:**

Prompt non-invasive imaging characterization allowed to understand the singular vascular abnormality and mini-invasive interventional radiology management resolved portal hypertension and variceal bleeding.

## Background

Anomalies of the portal venous system can be congenital or acquired, the latter being related to spontaneous thrombosis or iatrogenic alterations such as complications of perinatal catheterization of the umbilical vein (Corness et al. 2006; Carneiro et al. 2019). Congenital absence of the portal vein (CAPV) is associated with different degrees of porto-systemic shunts (Abernethy malformation (Rajeswaran et al. 2020)) with concomitant congenital malformations of the liver and cardiovascular system and related syndromes such as hepatic encephalopathy, hepatopulmonary syndrome and hepatorenal syndrome (Shen and Zhu 2008). Portal vein thrombosis (PVT) must be distinguished from CAPV: in particular when thrombosis occurs gradually, or during embryologic development, this can be very difficult. Differential diagnosis in favor of PVT is the presence of venous collaterals or secondary signs of portal hypertension, such as splenomegaly or ascites (Albers and Khanna 2019). Imaging provides a very accurate representation of the actual anatomic situation, but cannot always assess the evolution of events and establish an exact diagnosis.

The aim of this report is to present a singular case in a pediatric patient with malformation of the portal venous system characterized by unique imaging findings and to describe the interventional procedures applied in its successful management. Written informed consent was obtained from the patient for publication of this case report and any accompanying images.

## Case presentation

An 8-year-old female with no medical history, was referred to the Pediatric Intensive Care Unit due to severe hemorrhagic shock from esophageal varices bleeding. The child underwent blood transfusions and emergent gastroscopy with successful variceal ligation and hemodynamic stabilization.

On physical examination, she only presented an evident venous reticulum on her chest, without hepatosplenomegaly or other signs of portal hypertension (such as ascites, encephalopathy or confusion). Blood tests, including platelet and white blood cells count, liver function and coagulation, were within normal limits both at the admission and discharge.

**Fig. 1:**
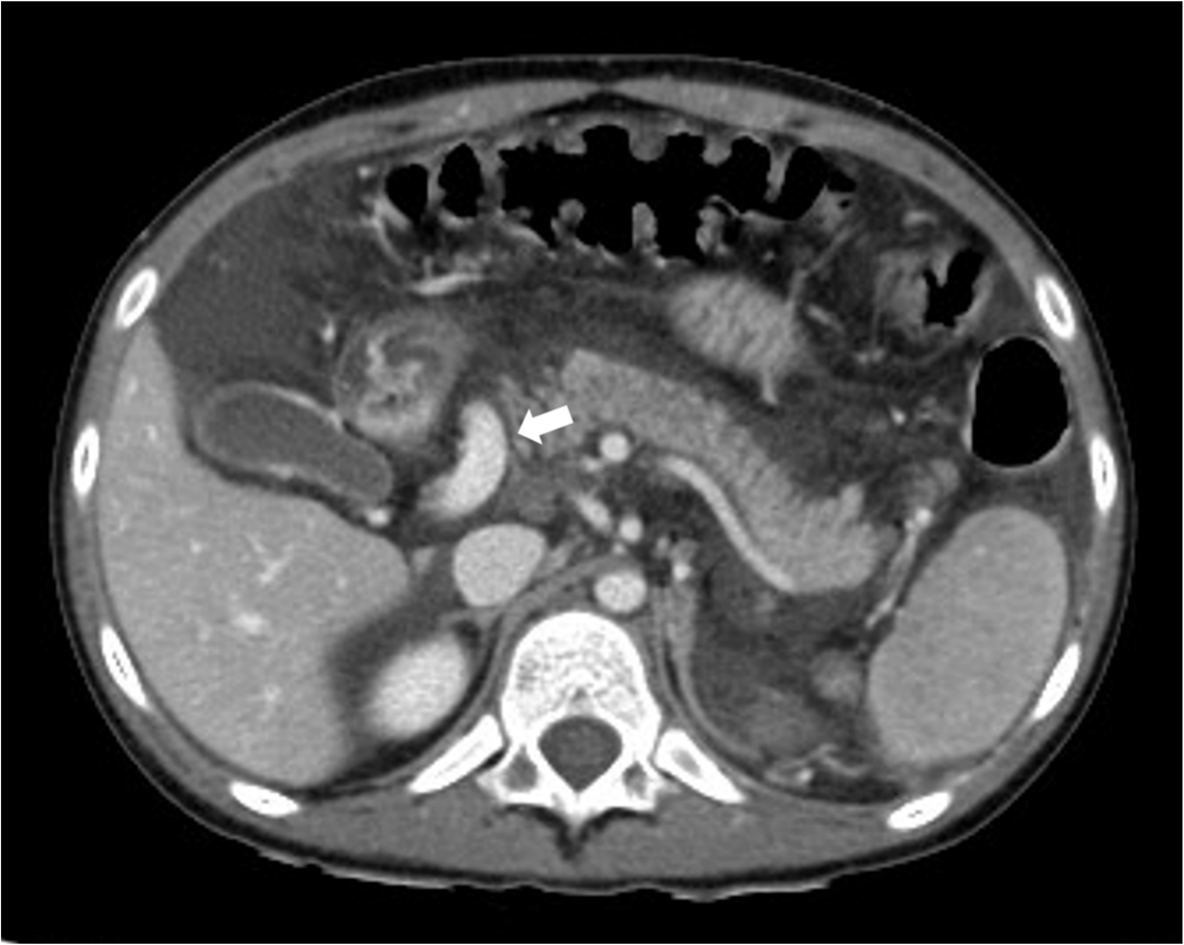
CTA of a 8-year old female who presentend with hematemesis due to variceal bleeding. Axial CT image in the portal venous phase shows an aberrant mesenteric retroduodenal venous collateral (arrow)

**Fig. 2:**
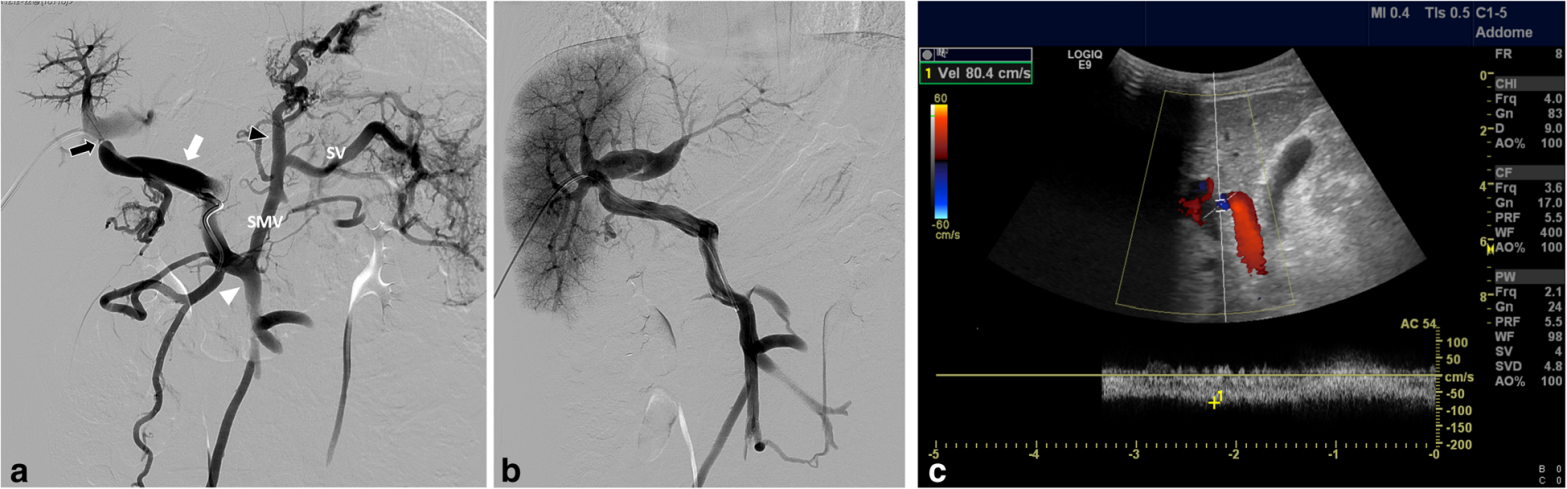
Images of percutaneous transhepatic portography before and after angioplasty and 1-month follow-up US. **a**) Venography of the splanchnic venous system performed after transhepatic catheterization of the venous collateral (white arrow) originating from the mesenteric vein (arrowhead) shows stenotic confluence with the right portal branch (black arrow); the splenic and mesenteric veins present hepatofugal flow and merge into a dilated gastric vein with varices (black arrowhead); no portal vein origin is detectable at its conventional anatomic site. **b**) Control venography of the splanchnic venous system performed after angioplasty shows restoration of hepatopetal flow with no opacification of the splenic and gastric veins and varices. **c**) After one month the confluence between the mesenteric venous collateral and the right portal branch presents a 5-mm caliber with a flow velocity of 80 cm/s. SMV, superior mesenteric vein; SV, splenic vein

Based on these findings and no liver disease history, abdominal Computed Tomography Angiography (CTA) and Doppler Ultrasound were performed to assess the liver parenchyma and vascularization. CTA showed normal liver morphology, volume and density; absence of splenomegaly and ascites. The extrahepatic portal vein was not detected at the expected site, and a single large mesenteric retroduodenal venous collateral (Fig. 1a) connected with the intrahepatic bifurcation of the right portal branch was observed. The anatomy of the intrahepatic portal venous system was substantially normal except for a dilated right portal branch. The splenic and superior mesenteric veins were patent with normal caliber, merging into a slightly dilated left gastric vein: a normal portal vein origin was absent. The enlarged gastric vein fed the esophageal varices. Doppler Ultrasound showed the presence of a stenosis at the confluence between the mesenteric venous collateral and the right intrahepatic portal branch, with a peak velocity of 150 cm/s and a pre-stenotic hepatopetal flow of 13 cm/s. No other vascular abnormalities such as arterio-venous fistulas or Abernethy shunts were noted. The biliary tree was not dilated; gall bladder and pancreas were normal. Based on the non-invasive imaging findings, a percutaneous transhepatic portography was indicated to better assess the morphology and hemodynamics of the splanchnic venous system. Under general anesthesia ultrasound-guided transhepatic catheterization of the right portal branch was performed with a 4 Fr coaxial introducer system (Neff Percutaneous Access Set, Cook Incorpo-155 rated, Bloomington, IN, USA). Percutaneous portography revealed a normal intrahepatic portal anatomy except for a dilated right portal branch. Under fluoroscopic guidance (Allura Xper FD20; Philips Healthcare, Best, the Netherlands) the mesenteric venous collateral was catheterized with a 4 Fr Simmons-shaped catheter (Cordis Corporation, Miami Lakes, FL, USA). Percutaneous venography of the splanchnic venous system confirmed the stenosis at the confluence between the mesenteric venous collateral and the right portal branch, with retrograde flow to the gastric vein and esophageal varices (Fig. 2a). Venous pressure in the mesenteric vein was above normal range (25 mmHg), suggestive of severe portal hypertension, with a trans-stenotic gradient of 18 mmHg. The 4 Fr introducer was exchanged with a 6 Fr vascular sheath (Merit Medical System, South Jordan, UT, USA) on a 0.035″ Amplatz guidewire (Cook Incorpo-155 rated, Bloomington, IN, USA). A 10-mm balloon (Mustang, Boston Scientific, Marlborough, MA, USA) angioplasty of the stenotic tract was performed and control venography showed an improved portal flow to the liver with restoration of the normal anterograde flow in the gastric and splenic veins (Fig. 2b). Final venous pressure in the mesenteric vein decreased to 14 mmHg; the same pressure was measured in the intrahepatic branches with no trans-stenotic gradient. The transhepatic tract was embolized using cyanoacrylate (Glubran 2, GEM, Viareggio, Italy) without intraprocedural complications and the child was discharged in good conditions after 1 week. Doppler Ultrasound performed 72 h, 1 month and 6 months after the procedure showed an adequate hepatopetal flow (50 cm/s) within the mesenteric venous collateral. The confluence between the collateral and the right portal branch presented a 5-mm caliber with a maximum velocity of 80 cm/s (Fig. 2c), with no residual hemodynamically significant stenosis. Intrahepatic portal venous flow was present, turbulent, with a mean velocity of 50 cm/s. The clinical benefit of the treatment was proven by the absence of varices at the 1-month followup gastroscopy and no bleeding episodes, with normal blood counts, during the 6-month follow-up.

## Conclusions

Developmental anomalies and acquired alterations of the portal venous system lead to a wide spectrum of uncommon clinical conditions. Herein, we describe a unique and singular case which has some clinical features common to PVT. Interestingly, in this case portal hypertension was not associated with ascites, hypersplenism or altered liver morphology: we hypothesize this was due to absence of liver disease and to the peculiar vascular anatomy, with spontaneous portal system decompression through an enlarged left gastric vein; although reduced by the stenosis, liver flow was probably enough. The case presents peculiar aspects suggesting a possible developmental anomaly: we hypothesize the persistence of the caudal ventral anastomosis of the right vitelline vein that may have compensated a portal vein agenesis or embryonic thrombosis. To our knowledge this vascular abnormality has been never reported with this clinical presentation.

Percutaneous transhepatic angioplasty led to the resolution of portal hypertension with no further risk of bleeding.

## Data Availability

The datasets used and/or analysed during the current study are available from the corresponding author on reasonable request.

## Abbreviations

CAPV: Congenital absence of the portal vein
PVT: Portal vein thrombosis
CTA: Computed Tomography Angiography
